# Editorial: Recent advances in genomics and oncogenomics for personalized medicine

**DOI:** 10.3389/fgene.2025.1635341

**Published:** 2025-07-02

**Authors:** Sulev Koks

**Affiliations:** Personalised Medicine Centre, Murdoch University, Perth, WA, Australia

**Keywords:** genomics, oncology, personalised medicine, genomic medicine, transcriptomics

This special issue on “Recent advances in genomics and oncogenomics for personalized medicine” is devoted to research reports focusing on the application of genomics in oncology. Genomics has existed for a considerable time, and its primary applications aim to improve cancer patient care, making it logical to dedicate this issue to the topic. All published papers have a direct implication of genomics, such as genomic profiling and mutation detection, or assess treatment efficacy concerning patients’ mutation profiles. As these papers illustrate, genomics is reshaping oncology and how we clinically manage our patients. Despite the progress made, several challenges remain and need to be addressed in future research endeavours.

Genomic stratification and mutation profiles vary across different populations. Similar to other multifactorial conditions, the frequencies of various mutations differ among populations; thus, clinical guidelines based on genomic profiles from one population may not be effective in others. More data on the genomic heterogeneity of these malignancies is essential. One approach to tackle this issue is to utilise large databases and cohorts from multiple populations. Additionally, identifying the ancestral population can enhance the power of this type of analysis.

The accurate diagnosis of cancer and its clinical management relies on genomic pathology. While histological typing remains important, an increasing body of data indicates that genomic profiling is vital for improved management and therapeutic response. This forms the cornerstone of the personalised medicine approach, with numerous studies suggesting the practicality of genomics-based clinical management. Cancers once deemed hopeless have been found to be curable following genomic analysis and precise genomic diagnosis. Although this can be challenging and may evolve into a significant diagnostic odyssey, our tools are advancing, and we have made notable progress with many types of cancer. Not only does the mutation itself pose difficulties, but its expressivity and penetrance also complicate the definition of the mutation and the accuracy of diagnosis. Moreover, challenges arise from the clinical description of the disease and the variation in treatment response. Therefore, genomics must be integrated into the diagnostic process as early as possible, and a multidisciplinary team should be involved from the start.

From a genomic perspective, the evolution of sequencing technologies has had a significant impact. Beginning with short-read sequencing and exome-focused or targeted gene-panel approaches, we have now entered an era where whole-genome sequencing is affordable for both short and long reads. Additionally, the speed of laboratory workflows has improved. Human whole-genome analysis now takes only days, and it is possible to obtain sequencing data in real time, making genomic information highly accessible. This implies that rather than simply getting a genome sequenced, the challenge now lies in the informatics pipeline. This encompasses not only the computing workflow but also our understanding of genome structure. Repetitive or transposable elements have long been labelled as junk DNA, with little relevance to clinical genomics. However, it is now widely recognised that the missing heritability resides within these repetitive elements, which were previously difficult to study before whole-genome sequencing became a commodity and easily accessible. Indeed, carcinogenesis, which has mainly been attributed to single-nucleotide variants, is now understood to primarily stem from the activity of transposable elements and the mutations these elements can cause. Transposable elements are responsible for more than half of cancer-related mutations, and they have been “unseen” by the most commonly used genomic technologies in the past. Improved access to whole-genome sequencing has changed that, and we have started making new discoveries regarding cancer genomics.

Cancer genomics involves more than just DNA analysis and mutation identification; it inherently includes RNA studies as well. Cancer research will benefit from whole transcriptome analysis, which can reveal splicing alterations or changes in gene expression levels, as well as the suppression or activation of genes directly implicated in carcinogenesis. Whole transcriptome data can uncover direct targets and assist in drug selection. It also aids in gene expression profiling or fingerprinting of the cancer, which is central to the molecular stratification of cancers. Cancer tissue is typically directly accessible, making it an ideal source for transcriptome profiling, especially when paired with normal tissue from the same individual.

After identifying the carcinogenic mutation and its impact at the RNA level, the next step is developing RNA-based therapeutics, such as antisense oligonucleotides or RNA transfer to replace the affected gene. These offer new and evolving opportunities to create truly personalised treatments for cancer patients based on genomic and transcriptomic information. Antisense therapeutics have made breakthroughs for rare diseases, and we will soon see similar success for personalised oncology.

Ultimately, and most importantly, to succeed in this progress and fully realise the opportunities genomics offers for oncology, we need greater involvement from patients and communities. This involvement begins with recognising that there are ways to integrate innovation into standard clinical care and to bridge the translational gap that prevents research-based discoveries from reaching the clinic. Patient and community involvement includes educating stakeholders and community members that many solutions are very close to clinical management and that they require just a small push or demand from the communities and funders. Genomics has the potential to offer treatment for every cancer patient, achievable when all community members are engaged and involved. Thanks to biology, we have seen improvements in cancer care and a reduction in the use of chemotherapy. Genomic medicine and gene-based therapeutics can render chemotherapy unnecessary, reducing its role in cancer management to the point of becoming history. We still have a long way to go, but let this special issue serve as a small step along this road. This special issue provides a broad overview of how genomics can be applied in oncology and how clinical management will evolve in the future.

